# Atypical Costochondritis: Complete Resolution of Symptoms After Rib Manipulation and Soft Tissue Mobilization

**DOI:** 10.7759/cureus.14369

**Published:** 2021-04-08

**Authors:** Javier Barranco-Trabi, Victoria Mank, Jefferson Roberts, David P Newman

**Affiliations:** 1 Department of Internal Medicine, Tripler Army Medical Center, Honolulu, USA; 2 Department of Rheumatology, Tripler Army Medical Center, Honolulu, USA; 3 Pain Management-Physiotherapy, Tripler Army Medical Center, Honolulu, USA

**Keywords:** costochondritis, inflammation, costosternal syndrome, parasternal chondrodynia, anterior chest wall syndrome, tietze syndrome, physical therapy, rib manipulation, soft tissue mobilization

## Abstract

Costochondritis is a self-limiting, poorly described, and benign condition that usually manifests as non-cardiac chest pain. The symptoms usually tend to resolve in a couple of weeks. Serious causes of chest pain should be ruled out prior to diagnosing costochondritis, as it is often a diagnosis of exclusion. Costochondritis that does not self-resolve is referred to as atypical costochondritis and is associated with high medical expenses and psychological burden on the patient.

In this report, we discuss the case of a 37-year-old healthy male patient who presented with a two-year history of intermittent pain along the right anterolateral rib cage without any history of trauma. After extensive diagnostic work-up by Cardiology and Gastroenterology, Physical Medicine and Rehabilitation (PM&R) got involved. The initial diagnosis of chest pain evolved into atypical costochondritis given the time course, physical examination findings of focal tenderness, along with normal laboratory values, electrocardiogram, and imaging studies. A multimodal approach was adopted for the treatment of this patient, including manipulative therapy to determine if regional interdependence was present, followed by instrument-assisted soft tissue mobilization (IASTM) and stretching to address the potential myofascial pain generators. After three appointments, there was complete resolution of morning pain and there was no pain upon examination.

This case highlights how osteopathic manipulation techniques (OMT) can be useful in the treatment of rib dysfunction, especially in atypical costochondritis. Further studies are required to expand our knowledge of costochondritis and physical therapy (PT) techniques, which would allow for early identification and effective treatment of the condition.

## Introduction

Costochondritis is a benign etiology of chest pain that is caused by inflammation of the costochondral joints. The chest wall pain, in most cases, is described as a dull, aching, sharp, or stabbing pain that varies in intensity. The pain is exacerbated by deep inspiration, upper extremity movements, and exercise [1]. Costochondritis is most likely associated with a history of recent strenuous activity or recent upper respiratory illness [2]. Inflammation can last from several weeks to several months [3], limiting the patient’s ability to work and perform activities of daily life. Costochondritis is also known as anterior chest wall syndrome, parasternal chondrodynia, and costosternal syndrome [4]. 

The differential diagnosis for non-cardiac chest pain is broad, and costochondritis should be differentiated from other causes of pain such as arthritis of sternoclavicular joint, fibromyalgia, herpes zoster of the thorax, painful xiphoid syndrome, slipping rib syndrome, Tietze syndrome, traumatic muscle pain, and overuse myalgia [3]. The diagnosis is made based on the exclusion of other causes of chest pain and the ability to reproduce the pain by palpation to the area [5]. 

The incidence and prevalence of costochondritis are not well established in the literature; however, costochondritis is a common diagnosis among patients with chest pain in the primary care clinician’s office and emergency department, most frequently described in Hispanic populations and females. Even though costochondritis is a frequent diagnosis related to chest pain, the etiology, treatment, and evolution of the disease are often poorly documented [1]. 

Treatment of costochondritis can involve pharmacological therapy, physical therapy (PT), or a combination of both. The drug of choice for costochondritis is nonsteroidal anti-inflammatory drugs (NSAID). The NSAID of choice is patient-specific and based on the provider's preference. It is important to discuss the adverse effects of NSAID use with the patient, such as the risk of gastritis. Severe or refractory costochondritis can be treated with PT. However, despite being a common diagnosis, the patients are not routinely referred to undergo PT [6].

The purpose of this case study was to describe the application of a sequenced musculoskeletal treatment approach in a patient with atypical costochondritis. Secondly, we also propose diagnostic and treatment recommendations that may serve to decrease the use of potentially unnecessary, costly, and invasive procedures while expediting access to effective care.

## Case presentation

A 37-year-old male patient presented with a two-year history of intermittent pain along the right anterolateral rib cage. The patient did not endorse a history of trauma. He had been waking up every morning with pain that lasted approximately 15 minutes. The pain was described as sharp and burning in nature and worsened with palpation along the costochondral cartilage between the eighth through 10th ribs and sternum. The pain resolved over time but returned while running or performing cardiovascular exercises. The patient reported that his pain ranged from 4/10 to 6/10 on the visual analog scale when present. While these morning symptoms did not affect his ability to perform activities of daily living or work, he was frustrated by the chronicity of symptoms and the lack of response to conservative measures.

Symptom onset was insidious in nature. The pain was initially noted on the right side of the chest wall, but there was a history of left-sided chest wall pain for several months that had subsequently resolved. He underwent a diagnostic workup by Cardiology, Gastroenterology, and Physical Medicine and Rehabilitation (PM&R). The initial diagnosis of chest pain evolved into costochondritis as reported by the gastroenterologist given the focal tenderness, absence of gastrointestinal alarm symptoms, normal laboratory test values, normal chest radiographs, and a lack of history of post-herpetic rash.

After two years of recalcitrant right-sided costochondritis, the patient was referred to the Interdisciplinary Pain Management Clinic (IPMC) for a trial intercostal nerve block. Pre-procedural pain was a 7/10 with palpation. The procedure involved injecting 4 ml of 0.5% ropivacaine and 8 mg of dexamethasone at the right T8-9 intercostal space. Post-procedure pain was reported to be 0/10, lasting for approximately one week. Upon reassessment three weeks later, the patient reported pain along the thoracic spine at the T8-9 level along with his baseline anterior costochondral pain. The patient was then referred to the IPMC physical therapist to investigate a potential rib component of his pain as demonstrated in a previous case study [7]. The patient’s primary goal was diagnostic clarity. His secondary goal was the resolution of his pain.

Physical therapy examination/evaluation

Physical evaluation revealed that the patient’s pain was localized to an area of the costochondral cartilage from the sternum to the 10th rib tip on the right side with deep palpation. The cervical spine was cleared after the patient demonstrated a full range of motion (ROM), a negative Spurling’s test, normal dermatomal sensation, and absence of myotomal weakness. Thoracic ROM was assessed in a standing position. The patient demonstrated full cardinal plane motion; however, combined thoracic extension and rotation to the right side reproduced his anterior chest pain.

Motion palpation testing

**Figure 1:**
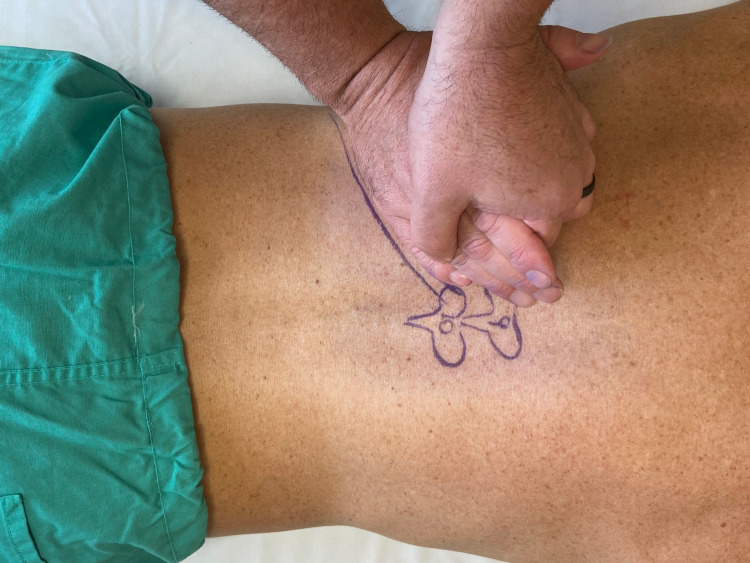
Rib spring maneuver applied to the 10th rib In prone, the examiner places the hypothenar area of the hand over the 10th rib angle. Force is applied in an anterior direction and then released. The test is positive if the pain is reproduced when the force is released. (Photograph: Newman DP. Rib Spring Maneuver Applied to the 10th Rib. Reproduced with permission from the author, 2021)

**Figure 2:**
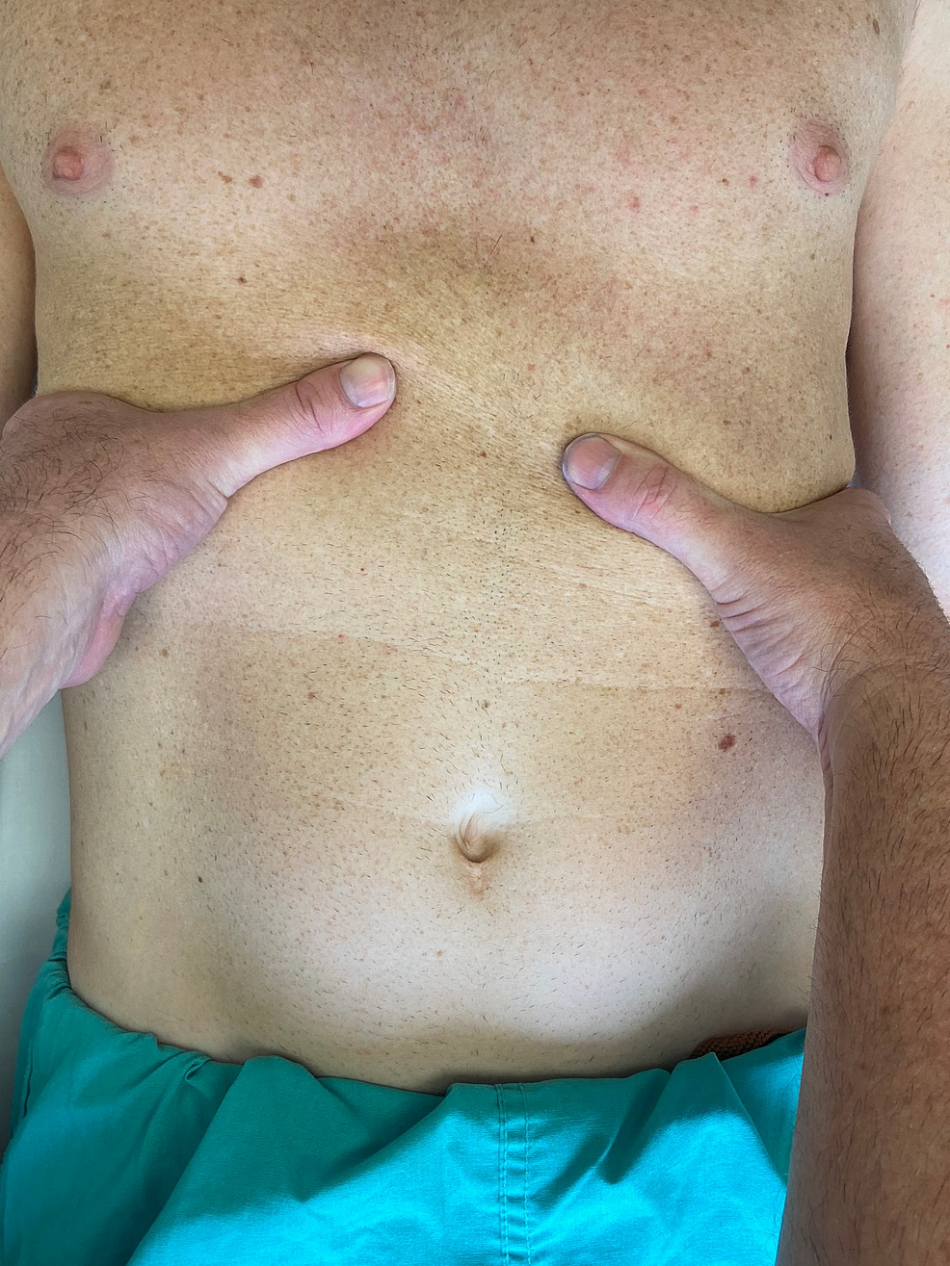
Assessment of rib excursion during inhalation In the supine position, the examiner places the thumb and index finger around the 10th ribs. The patient is instructed to inhale, and the examiner assesses for asymmetric motion (Photograph: Newman DP. Rib Spring Maneuver Applied to the 10th Rib. Reproduced with permission from the author, 2021)

Passive accessory intervertebral motion testing was performed in prone to the T1 through T12 spinous processes. Commonly utilized by physical therapists, this technique is considered to be useful in assessing segmental spinal hypomobility [8]. The restricted motion was assessed at T9 and T10 without pain reproduction. While in prone, provocation testing involving springing on the right 10th rib angle in an anterior direction did reproduce his pain (Figure 1). Rib mobility during inspiration was assessed in supine (Figure 2). The examiner placed fingers along the rib angles bilaterally and had the patient inhale deeply. The movement of the right ninth and 10th ribs was asymmetric compared to the left side.

Diagnosis/prognosis

The differential diagnosis specific to this patient’s symptoms upon initial evaluation to the IPMC included costochondritis, Tietze syndrome, intercostal neuralgia, slipping rib syndrome, rib dysfunction, myofascial pain syndrome, abdominal cutaneous nerve entrapment syndrome, fibromyalgia, and muscle strain. Tietze syndrome was ruled out based on the absence of swelling, erythema, and heat [9]. Given the immediate but short-term response to the intercostal trigger point injection, intercostal neuralgia and myofascial pain were high on the differential list. The patient’s signs and symptoms were consistent with the pathoanatomical diagnosis of costochondritis as the pain was reproduced by palpation over the costochondral cartilage; however, the presentation appeared atypical based on the consistent morning pain lasting 15 minutes, symptoms persisting for more than a year, and the location of structures involved [3]. Classically, the second through fifth costochondral junctions are involved in patients diagnosed with costochondritis [10,11]. As the pain was provoked with manual movement of the rib and thoracic spinal segment, it was positioned in a way that the faulty biomechanics of the rib and/or spine may increase load upon the cartilage, resulting in chronic pain.

The prognosis for complete pain resolution was poor to moderate based on the chronicity of symptoms and poor response to previous conservative measures. The prognosis would be adjusted based on the treatment response in each session.

Intervention

The proposed plan of care involved a sequenced approach of manipulative therapy to determine if regional interdependence was present, followed by instrument-assisted soft tissue mobilization (IASTM) to address the potential myofascial pain generators (Table 1). Regional interdependence is defined as unrelated functional impairments remote to the pain area or diagnosis that contributes to the problem [12]. Several case studies and a case series have described similar multi-modal treatment protocols that have been successful in treating costochondritis [6,7,12-15]. After each subsequent assessment, other therapeutic modalities would be included to both identify and treat contributory musculoskeletal faults.

**Table 1:** Overview of interventions applied and patient response per visit ROM: range of motion; OMT: osteopathic manipulation technique; IPMC: Interdisciplinary Pain Management Clinic

Visit	Patient pain presentation	Objective findings	Intervention	Patient response
1	Pain localized to the area of the right side of the rib cage from the sternum to the 10th rib tip; pain reported every morning upon waking and lasting 15 minutes (4-6/10 pain level)	Pain reproduced with deep palpation; combined thoracic extension and rotation to the right reproduces the pain; pain provocation with the springing of the right 10th rib angle; asymmetric motion of the ninth and 10th rib with inhalation	Ninth and 10th rib OMT; directional cupping along the costochondral cartilage from the 10th rib tip to the sternum/four 10-second periods	No pain reproduced with thoracic ROM after OMT; soreness after cupping (4/10 pain level); 50-75% reduction in AM pain the next morning
2	A 2/10 pain level along the costochondral cartilage	Mild pain reproduction with deep palpation; pain reproduced with combined thoracic extension and rotation to the right; negative rib provocation testing; symmetrical ninth and 10th rib motion with inhalation	Directional cupping performed along the ninth and 10th​​​​​​​ rib angle from the axillary line to the rib tip/four 10-second periods; instructed on latissimus dorsi and pectoralis major/minor stretching	No pain reproduced with thoracic ROM after OMT; soreness after cupping
3	Resolution of the morning pain; no pain upon examination	No pain with ROM; no pain with provocation testing; no pain with deep palpation	Discharge from IPMC	

**Figure 3:**
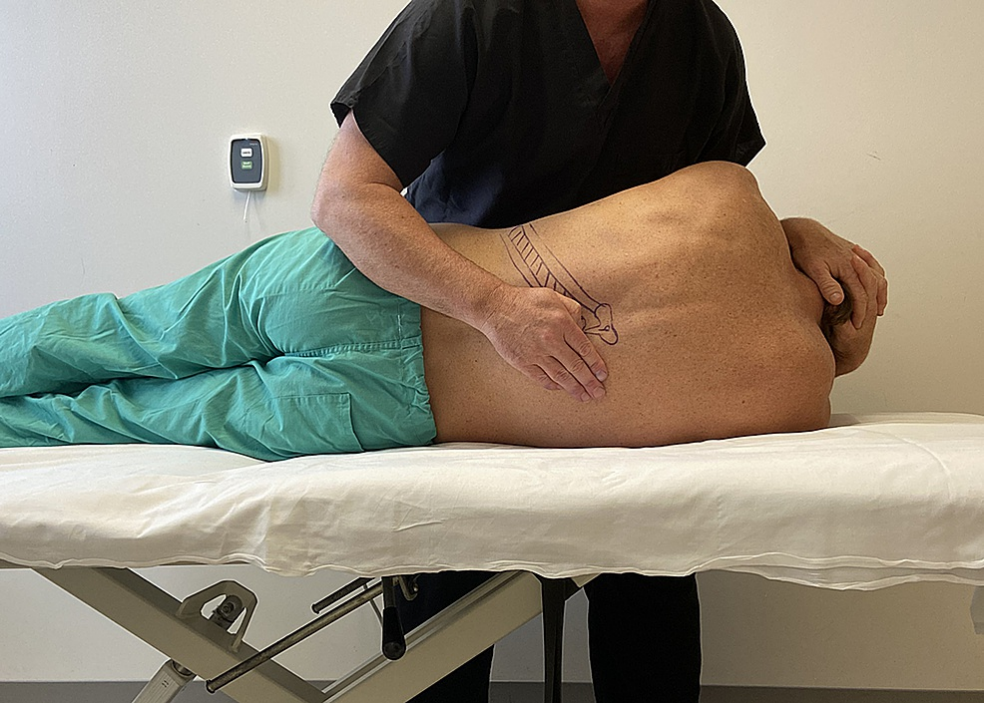
Rib manipulation technique - image 1 With the patient lying on their side, the 10th rib is identified, and the provider places the first metacarpal phalangeal joint of the palpating hand over the rib at the costotransverse joint and slides inferiorly to induce posterior rotation to the rib. The provider then moves the patient into a supine position. While the patient exhales, the provider imparts a high-velocity, low-amplitude force through the patient’s arms towards the examiner’s hand, which remains on their back (Photograph: Newman DP. Rib Manipulation Technique. Reproduced with permission from the author, 2021)

**Figure 4:**
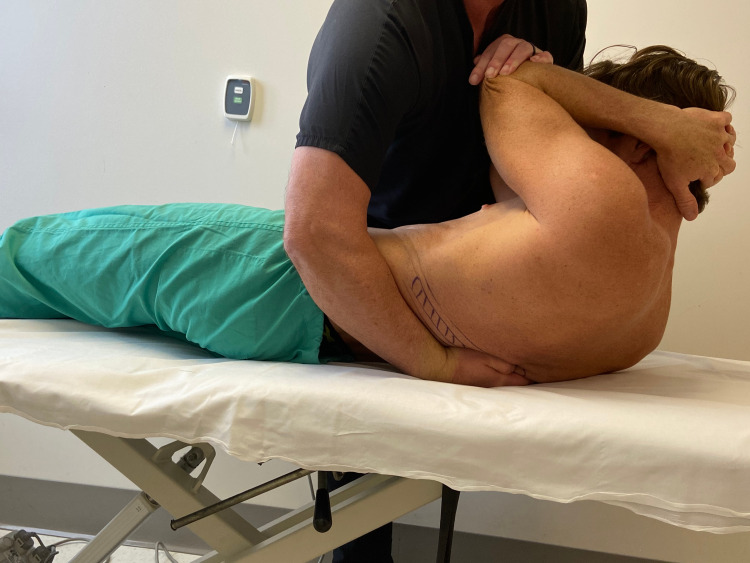
Rib manipulation technique - image 2 With the patient lying on their side, the 10th rib is identified, and the provider places the first metacarpal phalangeal joint of the palpating hand over the rib at the costotransverse joint and slides inferiorly to induce posterior rotation to the rib. The provider then moves the patient into a supine position. While the patient exhales, the provider imparts a high-velocity, low-amplitude force through the patient’s arms towards the examiner’s hand, which remains on their back (Photograph: Newman DP. Rib Manipulation Technique. Reproduced with permission from the author, 2021)

The patient was treated upon the initial evaluation with osteopathic manipulation techniques (OMT). OMT techniques have been shown to be effective in the treatment of rib dysfunction [14]. A posterior rotation force was applied to the right ninth and 10th rib separately with audible cavitation (Figures 3, 4). Upon reassessment of active thoracic ROM, the patient’s pain was not reproduced with combined thoracic extension and rotation to the right side.

Tenderness to deep palpation was still present; therefore, a trial of tissue mobilization over the area of the costochondral cartilage from the ninth and 10th rib tips to the sternum was performed. Described by Newman and colleagues (2020), this technique involved lubricating the skin with lotion and placing a 4-cm vacuum suction cup over the distal sternum. The cup is manually moved laterally to the lower rib tips and then back towards the sternum for a period of 10 seconds. After a short period of rest, cupping is continued for another three 10-second periods. The patient reported soreness after the treatment, rated at 4/10 with deep palpation. He was instructed to assess response to treatment the next morning and then follow up with the clinic.

**Figure 5:**
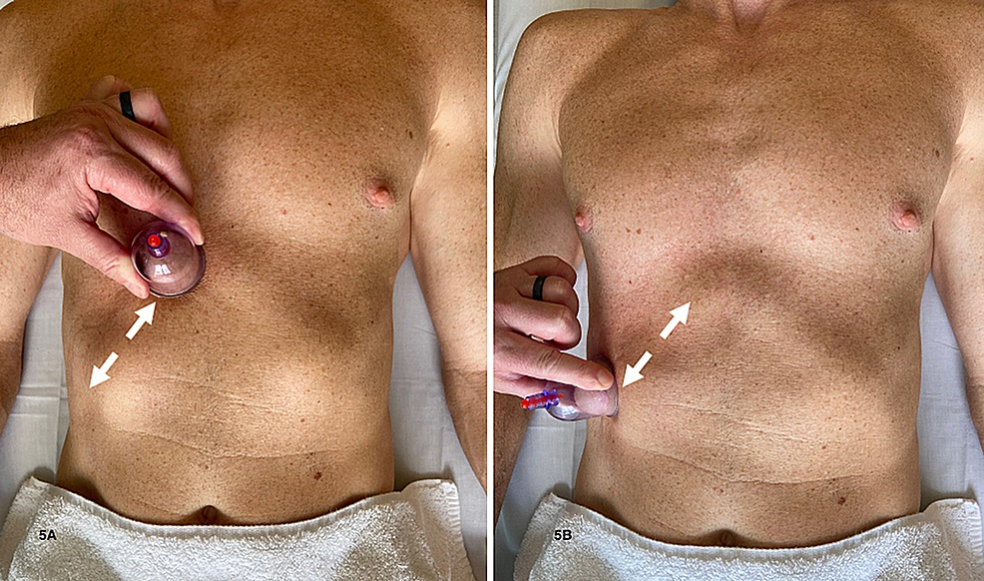
Directional cupping technique (A) Placement of the cup at the costochondral cartilage. (B) Movement along the cartilage to the ninth and 10th rib tips. After lubricating the skin with lotion, the 4-cm vacuum suction cup (KangZhu, China) was applied. The vacuum suction cup was manually moved back and forth across the skin with the goal of mobilizing soft tissues along the course of the costochondral cartilage from the sternum to the rib tips. This was repeated several times, taking breaks when the discomfort was too intense (Photograph: Newman DP. Directional Cupping Technique. Reproduced with permission from the author, 2021)

Upon reassessment the next day, the patient reported a 50-75% reduction in morning symptoms. There was still tenderness and mild bruising along the area of cupping. The pain level was reported as 2/10. A physical examination was performed. Upon thoracic ROM, combined extension and rotation to the right reproduced the patient’s pain. While there was restricted segmental mobility at T9/10 with motion testing, there was no reproduction of pain. Rib springing upon the ninth and 10th ribs did not reproduce pain. Rib motion during inspiration did not demonstrate any asymmetric motion compared to the left side. There was mild pain with deep palpation from the 10th rib tip to the sternum. Because provocation testing did not reproduce pain, OMT to the T9/10 segment was deferred. Instead, direction cupping was again applied to both address potential myofascial tightness and rule out regional interdependence [11]. The area of cupping was extended to incorporate the area from the sternum to the axillary line, in line with the ninth and 10th ribs on the right side.

The patient was instructed to perform latissimus dorsi and pectoralis major/minor stretching to maintain tissue mobility that improved following IASTM. In an eight-patient case series describing an impairment-based treatment program for costochondritis, latissimus dorsi and pectoral muscle tightness were observed in 50% and 100% of the patients, respectively [6]. Stretching has been shown to be efficacious in pain mitigation in patients with costochondritis compared to a control group [16]. On his third visit two weeks later, the patient reported resolution of the pain. While he did not perform the stretching exercises consistently, no pain was reported in the morning, with deep palpation, or with jogging. Thoracic ROM, spinal joint mobility, the springing of the ribs, and deep palpation did not reproduce pain. Symmetrical rib excursion was appreciated with palpation upon inspiration. Functional testing was performed by having the patient perform sit-ups. No pain was reproduced. The patient had met his care goals and was subsequently discharged from the IPMC.

## Discussion

Costochondritis is a self-limiting disease process that does not usually require any interventions; however, it can take up to one year for the condition to resolve [1]. The presentation of consistent chest pain every morning that abates after 15 minutes is atypical for costochondritis. Morning pain and stiffness are more likely associated with immune-mediated or rheumatological disorders of the musculoskeletal system, soft tissue injuries, autoimmune diseases, vasculitis, or inherited connective tissue disorders. The patient’s prognosis is based on the chronicity of symptoms. The time duration and resolution of symptoms are inversely related; therefore, the greater the duration of symptoms, the lower the likelihood of their resolution.

Atypical costochondritis is a diagnosis of exclusion. Life-threatening conditions such as acute coronary syndrome, acute aortic dissection, pulmonary embolism, tension pneumothorax, pericardial tamponade, and mediastinitis (e.g., esophageal rupture) need to be ruled out first before considering benign causes of chest pain [17]. There are expensive healthcare-associated procedures when evaluating for potentially life-threatening conditions, which include laboratory testing, radiographic imaging, and in some cases, referral to multiple specialists [18]. There are also psychological stressors that are inflicted on the patient due to the idea of having chest pain that could be a sign of a serious underlying condition.

This case report demonstrates the benefits of applying a sequenced musculoskeletal assessment and treatment approach on a patient with atypical costochondritis. The patient reported complete resolution of pain after three visits of treatment including OMT, directional cupping over the costochondral cartilage, and stretching after a two-year period of symptoms. Due to the complete resolution of symptoms in a short treatment course, this sequenced musculoskeletal approach may be an effective treatment option for patients suffering from atypical costochondritis.

The pathophysiology of costochondritis is poorly understood. Therefore, we are unable to determine the exact pathobiochemical reasons as to why OMT, directional cupping, and stretching help in the resolution of symptoms. Rabey (2008) postulates that the highly innervated costovertebral joints and surrounding structures or nociceptive afferent input to the ventral ramus from posterior spinal joints may account for a positive response to manipulative therapy [19]. These therapeutic approaches may reduce negative loading upon the joints or stimulate a beneficial neurogenic response, thereby reducing pain. Similarly, injured myofascial structures (i.e., pectoralis minor, intercostal muscles) local to the costochondral cartilage may be perceived as the pain generator when a load is applied [5]. Myofascial adhesion may be released and muscle tightness may be mitigated through directional cupping and stretching, respectively.

Though there are substantial benefits in PT techniques for pain management in costochondritis, there is usually a lag in time between the diagnosis and referral. The delay in referral to PT is unclear [2]. Some argue that costochondritis is a self-limiting condition, and by the time patients are seen by a physical therapist, the inflammation would have resolved on its own. We propose that atypical costochondritis refractory to conservative or “wait and see” management can have a beneficial response to OMT and tissue mobilization. This report adds to the literature about the effectiveness of OMT techniques and tissue mobilization in the treatment of atypical costochondritis. Allowing patients to reincorporate their activities of daily living while reducing the cost of healthcare is an important outcome that this case highlights.

The limitations of this case study include lack of external validity and the use of only one patient for the study of the methods and techniques applied. Therefore, the conclusion that these techniques may be beneficial for atypical costochondritis cases cannot be generalized to the wider population. Another limitation of our study is recall bias. Since our patient had been suffering from chest pain for two years, it is possible the patient was not able to recall all the pertinent information leading to the development of his pain and the disease progression.

## Conclusions

Costochondritis is a musculoskeletal condition that is poorly understood but has become the leading diagnosis related to non-cardiac chest pain. Early identification of this disease would likely reduce the unnecessary delay in definitive management and the need to undergo an expensive cardiac workup. OMT and tissue mobilization techniques may be effective therapeutic interventions in the successful treatment of patients with atypical costochondritis. More case studies are required to expand our knowledge of costochondritis and PT techniques, which would allow for early identification and efficacious treatment of the condition.
